# Catalytic Water Co-Existing with a Product Peptide in the Active Site of HIV-1 Protease Revealed by X-Ray Structure Analysis

**DOI:** 10.1371/journal.pone.0007860

**Published:** 2009-11-17

**Authors:** Vishal Prashar, Subhash Bihani, Amit Das, Jean-Luc Ferrer, Madhusoodan Hosur

**Affiliations:** 1 Solid State Physics Division, Bhabha Atomic Research Centre, Trombay, Mumbai, India; 2 Laboratoire de Cristallographie et Cristallogenèse des Protéines/Le Groupe Synchrotron, Institut de Biologie Structurale, Grenoble, France; University of Oulu, Finland

## Abstract

**Background:**

It is known that HIV-1 protease is an important target for design of antiviral compounds in the treatment of Acquired Immuno Deficiency Syndrome (AIDS). In this context, understanding the catalytic mechanism of the enzyme is of crucial importance as transition state structure directs inhibitor design. Most mechanistic proposals invoke nucleophilic attack on the scissile peptide bond by a water molecule. But such a water molecule coexisting with any ligand in the active site has not been found so far in the crystal structures.

**Principal Findings:**

We report here the first observation of the coexistence in the active site, of a water molecule WAT1, along with the carboxyl terminal product (Q product) peptide. The product peptide has been generated in situ through cleavage of the full-length substrate. The N-terminal product (P product) has diffused out and is replaced by a set of water molecules while the Q product is still held in the active site through hydrogen bonds. The position of WAT1, which hydrogen bonds to both the catalytic aspartates, is different from when there is no substrate bound in the active site. We propose WAT1 to be the position from where catalytic water attacks the scissile peptide bond. Comparison of structures of HIV-1 protease complexed with the same oligopeptide substrate, but at pH 2.0 and at pH 7.0 shows interesting changes in the conformation and hydrogen bonding interactions from the catalytic aspartates.

**Conclusions/Significance:**

The structure is suggestive of the repositioning, during substrate binding, of the catalytic water for activation and subsequent nucleophilic attack. The structure could be a snap shot of the enzyme active site primed for the next round of catalysis. This structure further suggests that to achieve the goal of designing inhibitors mimicking the transition-state, the hydrogen-bonding pattern between WAT1 and the enzyme should be replicated.

## Introduction

Human Immunodeficiency Virus (HIV) is the causative agent of Acquired Immunodeficiency Syndrome (AIDS) [1], [2]. Inhibitors of the viral enzyme HIV-1 protease (EC 3.4.23.16) are important components of Highly Active Anti Retroviral Therapy (HAART) for HIV/AIDS [3], [4]. The emergence of mutants of HIV-1 protease resistant to inhibitor action necessitates continuous improvement of existing drugs and also of design of new inhibitors. Understanding the catalytic mechanism and the structure and interactions of the transition state would contribute significantly in the development of novel inhibitors. Based on computational [5]–[8], biochemical [9]–[11] and structural results [12]–[16], two types of proposals have been made in the past for the catalytic mechanism: direct and indirect [reviewed in 17]–[18]. In the direct type, championed mostly by computational studies, the nucleophilic attack on the carbonyl carbon atom of the scissile peptide bond is directly by carboxyl oxygen atom of the catalytic aspartates. In the indirect type, the attack is by a water molecule [19]. The position and hydrogen bonding patterns from this water molecule at the time of attack are different in different proposals of the catalytic mechanism, and therefore knowing the location and interactions of nucleophilic water molecule would be a step in establishing the correct mechanism for this enzyme.

HIV-1 protease is a homodimeric enzyme in which the active site is located at the subunit interface, with each subunit contributing one aspartic acid to the catalytic center. The active site is covered on the top by two flaps, which become ordered into a closed conformation whenever a substrate or inhibitor is bound in the active site. During virus maturation, HIV-1 protease cleaves viral polyproteins at nine different sites of varying amino acid sequences. A water molecule found symmetrically hydrogen bonded to carboxyl oxygen atoms of both catalytic aspartates in the high resolution crystal structures of unliganded enzyme, (PDB Id 1LV1 and 2G69) is believed to be the nucleophile. This belief has been questioned [20] recently on the grounds that in the crystal structures of enzyme-ligand complexes, this water molecule has not been found to coexist with the ligand. Thus the location of nucleophilic water in the active site of HIV-1 protease is still an open question. In this respect, we have been pursuing crystallographic studies on active HIV-1 protease complexed with different substrate peptides [21]–[23]. We have been able to carry out such studies because of our discovery of closed-flap conformation of the enzyme in hexagonal crystals of HIV-1 protease even when the enzyme is unliganded [24]–[25]. Complexes with oligopeptide substrates could then be prepared by soaking these native crystals into aqueous solutions of the substrates. The chemical conditions, pH for example, of these solutions could be varied to try trapping the reactants at different stages of the reaction. In the present study, native crystals were soaked into solution of the substrate of amino acid sequence His-Lys-Ala-Arg-Val-Leu*-NPhe-Glu-Ala-Nle-Ser (where * denotes the cleavage site and NPhe & Nle denote p-nitrophenylalanine and norleucine, respectively) at pH 7.0. It was found that the full length substrate was cleaved at the specific cleavage site (Leu-p-nitro-Phe). The N-terminal product peptide (P product) had diffused out leaving behind only the C-terminal product peptide (Q product) still bound in the enzyme active site. A set of water molecules had moved into the region vacated by the P product peptide. One of these water molecules (WAT1) is optimally positioned to be the nucleophile. In this position, the water molecule does not accept any hydrogen bond through its lone pair and also is a donor in two strong hydrogen bonds, two features that contribute significantly towards activation of the water molecule for nucleophilic attack [26]. This position is shifted by about 1.4 Å from that observed in all unliganded structures of HIV-1 protease. The position WAT1 overlaps exactly the hydroxyl group of the picomolar transition-state mimic inhibitor KNI-272. Adachi et al. have suggested this hydroxyl oxygen of KNI-272 to be an ideal position for a water molecule to launch nucleophilic attack on the scissile peptide bond [27]. Thus the present report of HIV-1 protease product complex is the first observation of putative catalytic water coexisting with the product peptide. This structure further suggests that the transition-state-mimics, such as KNI-272, should be so designed that they bind the catalytic aspartates with a hydrogen-bonding pattern similar to that of WAT1.

## Results

### The Model of the complex

**Figure 1:**
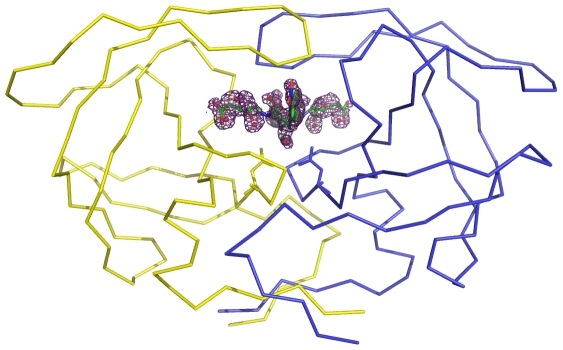
Difference density in the active site of HIV-1 protease/product complex. The enzyme is shown as a Cα trace. The side chains of catalytic aspartates are also drawn.

**Figure 2:**
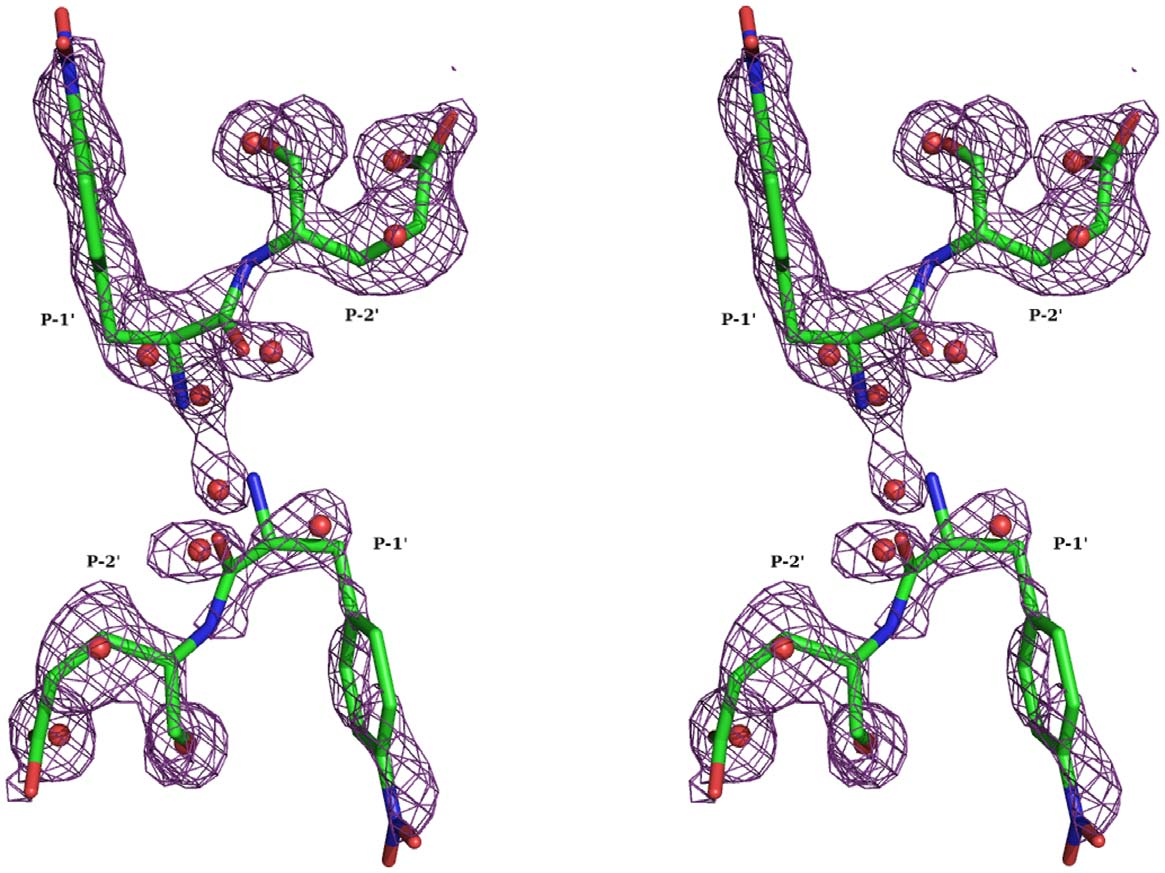
Fit of carboxy terminal product peptide and active site water molecules into 2Fo-Fc electron density. The electron density map is contoured at 1.0σ level. The carboxyl product peptide ( violetpurple) and the active site water molecules are shown in the two orientations.

HIV-1 protease tethered dimer used here contains a five residue linker, GGSSG, linking the N-terminus of second monomer to C-terminus of the first monomer [28]. Residues in the first monomer are numbered as 1–99 and those in the second monomer are numbered 1001–1099. Residues of the linker are numbered as 101–105. Crystal and intensity data statistics are given in Table 1. On refinement of the protein structure, difference density was found in the active site region of the enzyme (Figure 1), and this difference density represented the soaked-in substrate cleaved at the linkage connecting Leu and Nphe residues in the sequence. As per convention, residues in the C-terminal product (Q product) counted from the scissile bond were designated as P1'–P5', and those in the N-terminal product (P product) as P1–P6. The density for residues P1–P6 was very weak suggesting that the P product peptide had diffused out leaving behind only the Q product peptide still bound in the enzyme active site. A set of water molecules had substituted the P product peptide. Electron density for residues beyond P2' in the Q product was also very weak. The Q product and the water molecules were placed in two orientations, consistent with the pseudo-symmetry of HIV-1 protease active site. The lowest R_free_ was obtained when the occupancies for the two orientations were 0.7 and 0.3. The B-factor averaged over all atoms of the product peptide was 43.7 Å^2^ and 42.2 Å^2^ respectively for the two orientations. The electron density suggested that the side chains of few protein residues existed in multiple conformations in the crystal. Alternate conformations were modeled for the residues Val 82, Ile 84, Val 1082 and Ile 1084. There was no visible density in the 2Fo-Fc map for the linker region between residues 99 and 1001 of the tethered dimer under study, suggesting that the linker region was not ordered in the crystal. The final molecular model thus consisted of 1514 protein atoms, 181 water molecules and Q product peptide bound in two orientations with occupancies of 70% and 30% respectively. Conformationally, more than 90% of non-glycine residues were in the most favored regions of Ramachandran plot. The final refined 2Fo-Fc map for P1' p-nitro-phenylalanine and P2' glutamic acid residues and the active site water molecules in the two orientations are shown in Figure 2.

**Table 1:** Data collection and refinement statistics.

Space group	P6_1_
Unit cell parameters (Å)	a = b = 62.54,c = 81.99
Wavelength used (Å)	0.97945
Resolution (Å)	1.69 (1.72–1.69)*
Number of unique reflections	20251 (922)*
I/σ(I)	12.61 (3.35)*
R_merge_ (%)	8.2 (49.0)*
Completeness (%)	97.9 (94.1)*
Refinement statistics	
R_work_/R_free_ (%)	21.9 (30.2)*/25.6 (31.5)*
RMS deviations from ideal values	
Bond lengths (Å)	0.01
Bond angles (°)	1.5

*Data for highest resolution shell are given in the parenthesis.

### Protease- Q Product peptide interactions

**Figure 3:**
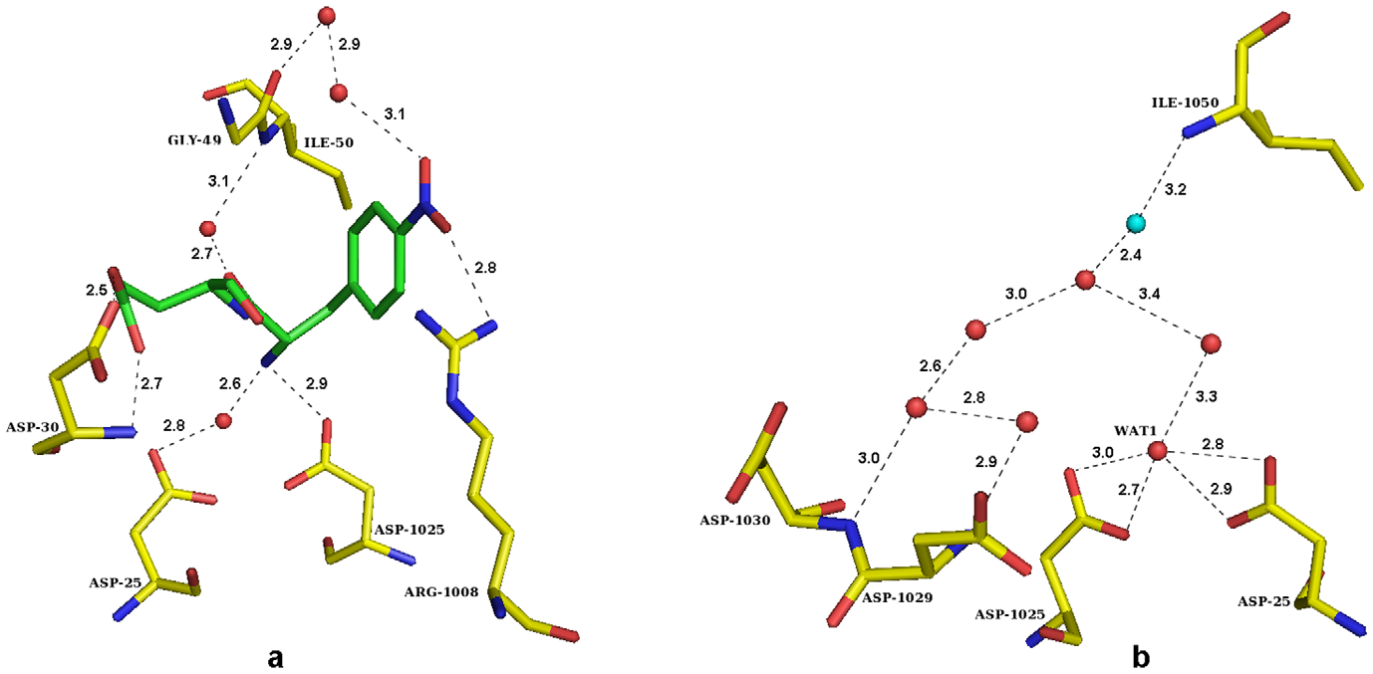
Hydrogen bonding interactions at the active site: a) between the protease and the P1'–P2' residues of the Q product peptide, b) between the protease and the water molecules. Possible hydrogen bonds are drawn as dotted lines, and the lengths are indicated.

Hydrogen bonding interactions between P1', P2' residues of the Q product and the protein residues in the active site are shown in Figure 3a. The Q product is held in the active site through 11 hydrogen bonds, some of which are through bridging water molecules. Terminal nitrogen of product peptide in both the orientations forms hydrogen bond to the outer oxygen (OD2) of ASP- 1025/ASP- 25 (Figure 3a). The side chain of P2' GLU forms hydrogen bonds with main chain amide nitrogen and side chain carboxyl oxygen of ASP- 30 or ASP- 1030 depending upon the orientation. One very well ordered water molecule forms the bridge between product peptide and the amide group of Ile 50/Ile 1050. One of the oxygens of P1' nitro group forms hydrogen bond with the Arg 8 while the other oxygen is bridged by two water molecules to the carbonyl oxygen of Gly 49.

### Water molecules in the active site

**Figure 4:**
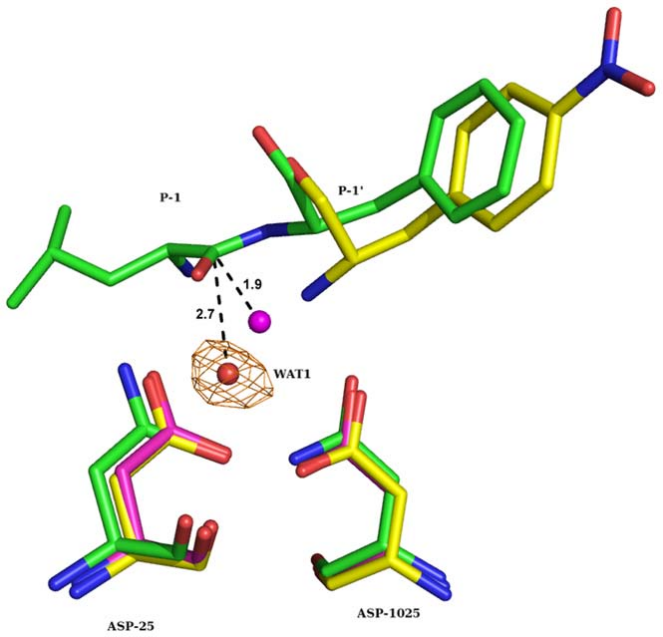
Relative positions of WAT1 and the modelled substrate in the active site: Diagram showing superposition of three structures: 1) present structure (yellow carbon), 2) unliganded HIV-1 protease (magenta carbon, PDB Id 1LV1) and 3) inactive HIV-1 protease/substrate complex (green carbon, PDB Id 1KJH). Water molecule observed in unliganded HIV-1 protease is also shown (magenta). The distances to the scissile carbon atom are indicated. SA OMIT density contoured at 3σ level is also shown for WAT1.

A set of water molecules had substituted the P product peptide. These water molecules are held in place through hydrogen bonds among themselves and also with the protein (Figure 3b). One of these water molecules, WAT1, which is within hydrogen bonding distance from the oxygens of both catalytic aspartates, may be of functional importance. The OMIT density for this water molecule is shown in Figure 4. WAT1 also makes a short hydrogen bond with the N-atom of the Q product peptide. WAT1 is shifted by about 1.4 Å from the corresponding water molecule coordinating both catalytic aspartates in the unliganded structures (PDB Id 1LV1 and 2G69). This water molecule is at an average distance of about 2.7 Å from the scissile carbon of the modeled substrate peptides (Figure 4).

## Discussion

### Position of attacking water molecule

**Figure 5:**
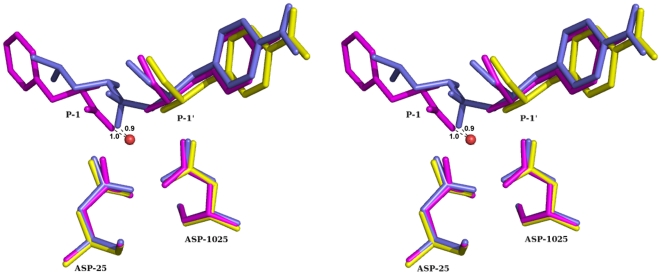
Structural comparison of present complex with tetrahedral intermediate complex [21] and product peptide complex [22]: Stereo diagram showing the ligand atoms at the catalytic centre along with catalytic aspartates. Protein Cα atoms are used in the structural superposition. WAT1 is within 1 Å from an oxygen atom in the newly generated gem-diol [21] or carboxyl group [22].

**Figure 6:**
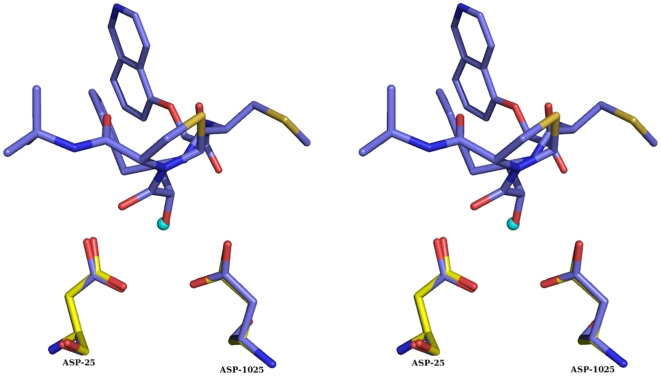
Position of the nucleophilic water molecule. Stereo diagram showing the overlap between the hydroxyl group (red) of transition-state mimetic inhibitor KNI-272 and WAT1 (cyan) of the present structure. The Q product peptide of the present structure is not shown. Note the perfect overlap of WAT1 and the hydroxyl oxygen of KNI-272.

**Figure 7:**
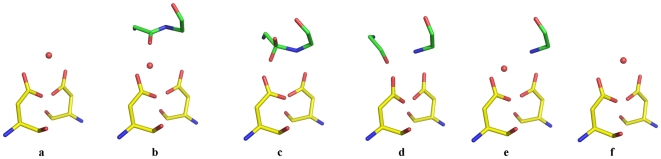
Proposed sequence of steps (a–f) in the cleavage of peptide bond by HIV-1 protease. Only main chain atoms of substrate peptide (green carbon) are shown. Each figure is based on the structure indicated: (a) catalytic water molecule bound symmetrically to the two aspartates (structure PDB Id 1LV1), (b) movement of catalytic water to position WAT1 with binding of substrate (modelled), (c) attack by WAT1 and formation of tetrahedral intermediate (Structure TI), (d) cleavage of the peptide bond with product peptides still bound in the active site (structure PDB Id 2NPH), (e) diffusion of P product out of and WAT1 into active site (present structure), and (f) release of Q product and movement of WAT1 into original position (PDB Id 1LV1).

In the hydrolysis reaction catalyzed by HIV-1 protease there are two substrates: 1) an oligopeptide of appropriate amino acid sequence and 2) the nucleophilic water molecule. At the start of the reaction, both these are bound in the active site leading to formation of Michaelis complex. At the end of the reaction, but before product release, the nucleophilic water is used up and hence should not be present in the active site. The presence of WAT1 in the active site places the present structure somewhere near the beginning steps of the reaction. Presently there is no crystal structure report of a Michaelis complex between active HIV-1 protease and substrate peptide. However, the present structure can be considered a close approximation to Michaelis complex since a part of the substrate peptide is present in the active site along with the water molecule. We have earlier reported the structures of HIV-1 protease complexed with two different substrate oligopeptides corresponding in amino acid sequence to the junctions RH-IN [21] and RT-RH [22] in the polyprotein substrate. While the substrate is converted into a tetrahedral intermediate in the complex with RH-IN, the RT-RH peptide is cleaved, with both product peptides still bound in the active site. The water molecule, WAT1, is at a distance of 0.9 Å from one gem-diol hydroxyl in the tetrahedral intermediate complex. Similarly WAT1 is at a distance of about 1.0 Å from one of the carboxyl oxygens in the product peptide complex (PDB Id 2NPH) (Figure 5). Because of these proximities, we suggest that the water molecule serving as the nucleophile in peptide bond hydrolysis does so from the position WAT1 observed in the present structure. Such a hypothesis would be consistent with the principle of least nuclear motion for chemical reactions [29]. To further explore this idea, we have investigated by molecular modeling, if the scissile peptide bond of a substrate bound in the active site would be accessible to WAT1 for attack. We have superposed separately the present complex with reported complexes between D25N inactive enzyme and two different substrate oligopeptides (PDB Id 1KJH and 3BXR) [30], [31]. Using only protein Cα atoms for structural superposition, equivalent positions of the substrate molecules were derived. Figure 4 shows the derived positions relative to WAT1. It is clear that the scissile peptide bond is optimally accessible to WAT1 for nucleophilic attack, the WAT1…C-O and WAT1…C-N angles being 69° and 104° respectively. Further, the distance of WAT1 to the scissile carbon atom is 2.7 Å, which is reasonable for a nucleophilic attack. Figure 4 also shows the position of the catalytic water observed in the structure of unliganded HIV-1 protease (PDB Id 1LV1). The separation of this water molecule from the scissile carbon atom is only 1.9 Å, which is too short a distance for the water molecule to stay in this position along with the substrate. Unlike in unliganded structures, the position of WAT1 is asymmetric with respect to the catalytic aspartates. WAT1 forms two short hydrogen bonds to outer and inner carboxyl oxygens of ASP- 25 and ASP-1025 respectively. Further, WAT1 does not accept any hydrogen bond and is a donor (see below) in two strong hydrogen bonds with catalytic aspartates. Both these features should increase the nucleophilicity of WAT1 [26]. From all these considerations, WAT1 appears to be a reasonable position for the water molecule from where nucleophilic attack takes place during bond breakage. This hypothesis is also consistent with the structure of HIV-1 protease/KNI-272 complex reported recently [27]. KNI-272 is one of very few highly selective and potent inhibitors of HIV-1 protease with a picomolar inhibitory constant. The high potency is suggested to be due to its pre-organized rigid structure that very closely resembles the transition state. The structure of the complex has been determined to very high resolution using X-ray and neutron diffraction techniques. According to the authors of this study, the position of the hydroxyl group in the hydroxymethylcarbonyl part of KNI-272 is ideal to mimic the location of the attacking water molecule in catalysis. Figure 6 shows the superposition of the present structure with the HIV-1 protease/KNI-272 complex mentioned above (PDB Id 3FX5). It is very interesting that WAT1 perfectly overlaps the hydroxyl group of KNI-272 in the complex. Since this overlap guarantees the adherence to the principle of least nuclear motion, KNI-272 is a very potent inhibitor of HIV-1 protease. In addition to hydrogen bonds to catalytic aspartates, WAT1 is hydrogen bonded to the terminal N atom of the Q product peptide. Once the Q-product leaves the active site, WAT1 will move back to the position observed in the structures of unliganded HIV1-protease. The relative positions of the substrate, nucleophile and catalytic aspartates at different stages of the cleavage reaction, according to our proposal, are shown in Figure 7 (a–f).

### Protonation state of catalytic aspartates

In the process of inhibitor design, it is important to both structurally mimic the transition state intermediate and to maximize interactions between the inhibitor and the catalytic aspartates. In this context, it is essential to know the protonation states of the catalytic aspartates so that appropriate functional groups are chosen in the inhibitor being designed. Even though hydrogen atoms are not located in the present study, the observed strong hydrogen bonds involving the catalytic aspartates provide a clue to the protonation states of the aspartates. Only O—O/N separation shorter than 2.8/2.9 Å are considered as definite hydrogen bonds [32]. There are four such distances at the catalytic centre in the present structure: i) ASP-25 OD1…ASP-1025 OD1, ii) WAT1… ASP-25 OD1, iii) WAT1…ASP-1025 OD2 and iv) ASP-1025 OD2…N-terminus of P1' residue. The angle ASP-25 OD2 –WAT1- ASP-1025 OD1 is 101° which is very close to the H-O-H angle (104°) in a water molecule indicating that in the hydrogen bonding to the water molecule WAT1, the aspartate oxygens act as acceptors. Since the substrate is already cleaved, N-terminus of P1' residue is already protonated and it would be a donor in the hydrogen bond with ASP-1025 OD2 atom. Thus the aspartic dyad is monoprotonated with the proton shared between inner oxygens of the two aspartates. We therefore suggest that just prior to the formation of the transition state the aspartates are in this state of protonation. Since on inhibitor binding the protonation state is not likely to change, the hydrogen-bonding group should be chosen appropriately on the inhibitor to maximize interactions with aspartates in this state of protonation.

### Effect of pH on conformation and interactions from ASP-25 and ASP-1025

HIV protease is known to be active over a wide range of pHs. In our earlier study of the crystal structure of HIV-1 protease complexed with the undecapeptide substrate (His-Lys-Ala-Arg-Val-Leu*-NPhe-Glu-Ala-Nle-Ser) at a pH value of 2.0, the substrate bound in the active site had transformed into a tetrahedral intermediate through nucleophilic attack by a water molecule [21]. In contrast, in the present study carried out at pH 7.0, the substrate molecule of the same sequence is found cleaved at the correct scissile bond, and the N-terminal P product peptide has diffused out of the enzyme active site. The conformations and interactions of the catalytic aspartates at the two pHs are compared in Tables 2 and 3. The changes in the conformations around main-chain and side-chain torsions of the two aspartates are very small, but these small changes have synergistically caused differences in the interaction distances, which could be significant. The hydrogen bonds from inner oxygen (OD1) atoms to N atom of corresponding Gly-27/1027 residues have become longer for both aspartates at pH 7.0. The distance between the two inner oxygen atoms, on the other hand, has changed in the opposite direction, that is, to shorter value, at pH 7.0. If the length of a hydrogen bond is assumed to reflect its strength, the changes in lengths mentioned above appear to preserve the total hydrogen-bonding ability of each OD1 atom. There is a significant change in the virtual dihedral angle OD2 (25)-OD1 (25)-OD1 (1025)-OD2 (1025), which is a measure of the co-planarity of the two aspartic acid side chains [15]. The two side chains tend toward being more co- planar at pH 7.0. There also appears to be a correlation between the co-planarity of the two aspartates and the strength of the hydrogen bond between the OD1 atoms of catalytic aspartates; the higher co-planarity leading to stronger hydrogen bond. In the structure of HIV-1 protease product complex [22] determined at a pH of 6.2 the aspartates are more co-planar with a virtual dihedral angle of 22° while the OD1..OD1 distance of the postulated hydrogen bond is only 2.3 Å.

**Table 2:** Comparison of conformation of catalytic aspartates in the structures at pH 2.0 and pH7.0.

Parameter compared	pH	χ_1_ (°)	χ_2_(°)	Φ(°)	Ψ(°)
**Conformation of Residue ASP-25**	2.0	−173	−25	−126	−85
	7.0	−171	−14	−120	−86
**Conformation of Residue ASP-1025**	2.0	−175	166	−116	−84
	7.0	−173	173	−121	−83

**Table 3:** Comparison of interactions of catalytic aspartates in the structures at pH 2.0 and pH7.0.

Parameter compared	pH 2.0	pH 7.0
**Interaction distance ASP-25 OD1- GLY-27 N**	2.8Å	2.9 Å
**Interaction distance ASP-1025 OD1- GLY- 1027 N**	2.7 Å	2.9 Å
**Interaction distance ASP-25 OD1- ASP-1025 OD1**	3.0 Å	2.7 Å
**Virtual Dihedral angle OD2(25)-OD1(25)-OD1(1025)-OD2(1025)**	78°	56°

### Product release

The patterns of product inhibition are dependent on the enzyme mechanism. Based on product inhibition and solvent isotope effects, in the cleavage reaction by HIV-1 protease the product peptides are proposed to be released in an ordered manner, with the P product peptide released first [11]. The presence of only carboxyl terminal product in the present structure is consistent with this expectation. The Q product peptide is tending to diffuse out of the active site, although more slowly, since the distance between Cα atom of P1' residue and Cγ atom of distal aspartate has increased from 5.0 Å in the tetrahedral intermediate structure [21] to 5.5 Å in the present structure (Figure 5). It is interesting that it is the N-terminal P product which is bound when active HIV-1 protease is cocrystallised with a constrained hexapeptide [31]. This difference may be due to the different approach taken for preparing crystalline enzyme/substrate complex. The constrained hexapeptide is cleaved during cocrystallisation, and from among the two products released into solution the P product is selectively bound in the active site because of its increased hydrogen bonding ability coming from the newly formed carboxyl group. Similarly, on cocrystallisation, the presence of an amino group in the product peptide PIV-CONH_2_ resulted in binding of PIV-CONH_2_ in the active site of HIV-1 protease, in an unexpected mode [13], [31].

### Conclusion

Native crystals of active tethered HIV-1 protease were soaked in an undecapeptide substrate solution at pH 7.0. Three dimensional crystal structure, determined to 1.69 Å resolution shows that the Q product peptide generated within the crystal is still bound in the active site of HIV-1 protease along with a set of water molecules. One of these water molecules, WAT1, which is activated through hydrogen bonds to catalytic aspartates, is located at a distance of 2.7 Å along the direction perpendicular to the scissile peptide bond. Assuming the present structure to be a close approximation to Michaelis complex, we propose that the incoming substrate pushes the nucleophilic water from the position observed in the unliganded protease to the WAT1 position from where it attacks the scissile peptide bond. Once the Q product also diffuses out, the catalytic water molecule can move back to the position observed in unliganded structures of HIV-1 protease. Comparison of geometries at the catalytic centre shows systematic changes in the conformation and interactions of catalytic aspartates at pHs 2.0 and 7.0. The structure reported here also suggests that in the design of effective inhibitors of HIV-1 protease, it is important to duplicate the hydrogen-bonding pattern of WAT1 with catalytic aspartates.

## Materials and Methods

### Protein expression, crystallization and soaking

HIV-1 protease tethered dimer used in the present study contains a five residue linker, GGSSG, covalently linking the two monomers [28]. Expression, purification and crystallization of HIV-1 protease tethered dimer followed the procedures reported earlier [24]–[25]. Briefly, BL21 (DE3) cells with HIV-1 protease tethered dimer insert carrying plasmid were grown at 37°C to an O.D_600_ of 0.6. The protease expression was induced by adding 1mM IPTG. Two hours after induction, cells were harvested and lysed using sonication to prepare the inclusion bodies. Inclusion bodies were thoroughly washed with Tris EDTA buffer and protein was extracted in denatured form with 67% acetic acid. Extract was diluted and dialyzed overnight against water. This was followed by dialysis against refolding buffer of pH 6.5, containing 20mM PIPES, 100mM NaCl, 1mM dithiothreitol and 10% Glycerol.

The cloned insert contains 57 extra codons in the beginning, which is a part of N-terminal polyprotein of *pol* gene. Therefore the inserted gene product is a 29 kDa precursor protein, containing natural cleavage site for HIV-1 protease, which after self cleavage results in a mature protein of 22 kDa.

Crystals were grown by hanging drop vapour diffusion method. Equal volumes of Protein (5 mg/ml in 50 mM sodium acetate, pH 4.5, containing 1 mM dithiothreitol) and reservoir solution (1% saturated ammonium sulfate, 200 mM sodium phosphate, and 100 mM sodium citrate at pH 6.2) were mixed on a cover slip and sealed over the reservoir well at room temperature.

The 11-residue substrate peptide of amino acid sequence His-Lys-Ala-Arg-Val-Leu-NPhe-Glu-Ala-Nle-Ser was synthesized at the National Institute for Research in Reproductive Health, Parel, Mumbai, by using an automatic peptide synthesizer. The peptide was dissolved in water to prepare a 5 mM stock solution. This stock solution was diluted 5-fold into the reservoir solution (pH 7.0) to prepare the soaking drop. Protease crystal was transferred first to a fresh reservoir solution (pH 7.0) drop to wash the crystal and then to the soaking drop using a cryoloop. The cover slip was inverted and sealed over the same reservoir well in which crystals had been grown.

### X-ray data collection and refinement

At the end of 72 h of soaking at room temperature, the crystal was equilibrated in the cryo-protectant (25% glycerol and 75% reservoir buffer) before flash freezing, for exposure to X-rays on the FIP-BM30A beam line [33]. The crystals diffracted to 1.69Å resolution. The diffraction data were indexed, integrated, and scaled by using the computer program XDS [34].

Computer program Phaser [35]–[36] from CCP4 suite was used to obtain molecular replacement solution using the structure 1LV1 [25], [37] as the search model. The structure was refined in Crystallography and NMR System (CNS) by using standard simulated annealing protocols and the amplitude-based maximum likelihood target function [38]–[39]. A test set containing 5.0% of randomly chosen reflections were reserved for determination of R_free_ [40], which is an indicator of gainful refinement. Occupancies of ligand molecules in the two orientations were systematically varied, in steps of 0.1, subject to the constraint that their sum be 1.0. Electron density maps of all types were calculated using CNS. All interactive model building and molecular superpositions were carried out using the graphics software O [41]. Structural comparisons are based on superpositions of protein Cα atoms. All figures were drawn using program Pymol [42]. Atomic co-ordinates and structure factors have been deposited in the Protein Data Bank under the PDB Id 2WHH.
